# The Comparative Analysis of Two RT-qPCR Kits for Detecting SARS-CoV-2 Reveals a Higher Risk of False-Negative Diagnosis in Samples with High Quantification Cycles for Viral and Internal Genes

**DOI:** 10.1155/2022/2594564

**Published:** 2022-07-05

**Authors:** Roberto Luraschi, Carlos Barrera-Avalos, Eva Vallejos-Vidal, Javiera Alarcón, Andrea Mella-Torres, Felipe Hernández, Ailen Inostroza-Molina, Daniel Valdés, Mónica Imarai, Claudio Acuña-Castillo, Felipe E. Reyes-López, Ana María Sandino

**Affiliations:** ^1^Centro de Biotecnología Acuícola, Facultad de Química y Biología, Universidad de Santiago de Chile, Santiago, Chile; ^2^Centro de Nanociencia y Nanotecnología CEDENNA, Universidad de Santiago de Chile, Santiago, Chile; ^3^Facultad de Medicina Veterinaria y Agronomía, Universidad de Las Américas, Santiago, Chile; ^4^Department of Biology, Faculty of Chemistry and Biology, University of Santiago de Chile, Santiago, Chile; ^5^Department of Cell Biology, Physiology and Immunology, Universitat Autònoma de Barcelona, Bellaterra, Spain

## Abstract

The early detection of severe acute respiratory syndrome coronavirus 2 (SARS-CoV-2) using the real-time quantitative polymerase chain reaction (RT-qPCR) as a gold-standard molecular tool has allowed to test and trace the viral spread and the isolation of COVID-19-infected patients. The detection capacity of viral and internal genes is an essential parameter to consider and analyze during the assay. In this study, we analyze the performance of the two commercial RT-qPCR kits used in Chile, TaqMan™ 2019-nCoV Control Kit v1 (Thermo Fisher) and MaxCov19 (TAAG Genetics), for the COVID-19 diagnosis from nasopharyngeal swab samples (NPSs). Our results show a lower sensitivity of the TAAG kit compared to the Thermo Fisher kit, even in the detection of SARS-CoV-2 mutations associated with its variants. This study reinforces the relevance of evaluating the performance of RT-qPCR kits before being used massively since those with lower sensitivity can generate false negatives and produce outbreaks of local infections.

## 1. Introduction

The COVID-19 pandemic has profoundly collapsed health systems in the world. Since it was declared a pandemic by the World Health Organization (WHO) on March 11, 2020 [1], more than 6.2 million people have died to date [2]. Although statistics currently indicate a decrease in infections and deaths in some countries with an increase in the vaccinated population [3], the appearance of new variants or genetic changes in the virus can undermine the protection provided by vaccines, increase infections, and generate sprouts locally and globally. Thus, it is crucial to keep up a continuous search and testing to detect new possible infections by SARS-CoV-2. One of the main ways to control its spread is the early and effective detection of positive cases. For this, the molecular technique of real-time polymerase chain reaction (RT-qPCR) has been widely used and recommended for the diagnosis of COVID-19 disease [4]. There are a series of commercial RT-qPCR kits available to carry out this technique in a massive way on the market. These allow the detection of different target genes of SARS-CoV-2, such as envelope (E), nucleocapsid (N), spike (S), open reading frame 1ab (ORF1ab), or RNA-dependent RNA polymerase (RdRp), depending on the type of manufacturer [5]. However, important differences in the detection efficiency could compromise the kit performance and diagnosis. For example, the Gerbion GmbH & Co. diagnostics kit showed a detection efficiency of 49%, while the kit from SolGent Co., Ltd. manufacturer showed a detection efficiency of 92% in SARS-CoV-2 detection [6]. On the other hand, a study in India showed differences of up to 21% between the RT-qPCR kits used for the COVID-19 diagnosis [7].

In this study, we evaluate the performance of the RT-qPCR TaqMan 2019-nCoV Assay Kit v1 (Thermo Fisher) and MaxCov19 kit (TAAG Genetics), widely used for the detection and diagnosis of SARS-CoV-2 in NPSs of patients from the population of Santiago, Chile. We compared the Cq values for the detection of internal reference and viral genes. We found that Cq values are directly related to the SARS-CoV-2 detection efficiency and diagnosis. The results indicate a lower sensitivity of the TAAG kit than the Thermo Fisher kit in detecting SARS-CoV-2 and its impact on the analysis and detection of SARS-CoV-2 variants. Our study emphasizes that the lower SARS-CoV-2 detection of an RT-qPCR kit could compromise the diagnostic and lead to uncontrolled spread.

## 2. Materials and Methods

### 2.1. Patient Samples

Nasopharyngeal swab samples (NPSs) were collected in the hospitals that belong to the Central Metropolitan Health Service (SSMC) of Santiago, Chile. The swab samples were preserved and transported in a CITOSWAB® transport kit (Reference code. 2118-0015; Citotest Labware Manufacturing Co., Ltd, Jiangsu, China). All the samples were processed before the first 24 hours after the sampling collection.

### 2.2. RNA Extraction

According to the manufacturer's instructions, total RNA extraction was done using the Pure RNA Extraction Kit (TAAG Genetics, Santiago, Chile. Reference Code: TAGGE01003). The extracted RNA was used immediately for SARS‐CoV‐2 detection by RT‐qPCR.

### 2.3. RT-qPCR Amplification Using Thermo Fisher Kit

The detection of SARS-CoV-2 was carried out using the ORF1ab gene, N gene, and S gene probe (TaqMan™ 2019nCoV Assay Kit v1 (Thermo Fisher Scientific, Reference code. A47532) using a one-step strategy. Positive control probes for viral genes and the RNase P internal reference gene (TaqMan™ 2019-nCoV Control Kit v1; Thermo Fisher Scientific, Reference code. A47533) were included and assessed in the PCR plate. The polymerase from TaqMan™ Fast Virus 1-Step Master Mix (Applied Biosystems™, Reference code. 44-444-36) was included in each reaction. The reactions followed the manufacturer's instructions with some modifications. Briefly, the reaction mix contained 5 *μ*l of TaqMan™ Fast Virus 1-Step Master Mix 4X, 1 *μ*l of viral gene assay 20X, 1 *μ*l of RNase P assay 20X, 11 *μ*l of nuclease-free water, and 2 *μ*l of total RNA extracted from the NPSs. The thermal amplification conditions include the reverse transcription at 50°C for 5 minutes, predenaturation at 95°C for 20 s, followed by 45 cycles at 95°C for 3 seconds and 60°C for 30 seconds. All the RT-qPCRs were performed on the Agilent AriaMx Real-Time PCR System (Agilent Technologies, Reference code. G8830A). Data were extracted using the Agilent AriaMx software.

### 2.4. RT-qPCR Amplification Using TAAG Kit

The detection of SARS-CoV-2 was made following a one-step strategy using the RT-qPCR MaxCov19 (TAAG Genetics, Reference code. TAGK01052). This assay was carried out using the N1 and E gene probes (loading 5 *μ*l of total RNA extracted from NPSs per reaction), with some modifications. In brief, 2 *μ*l of total RNA extracted from NPSs and 3 *μ*l of nuclease-free water were loaded in each reaction. Positive control probes for viral genes and RNase P (internal reference gene) were included and assessed individually in the PCR plate. All the RT-qPCRs were performed on the Agilent AriaMx Real-Time PCR System (Agilent Technologies, Reference code. G8830A). Data were extracted using the Agilent AriaMx software.

### 2.5. PCR Efficiency and Limit of Detection (LoD)

To establish PCR efficiency and LoD for internal reference and viral gene probes assessed for TAAG and Thermo Fisher kits, we ran RT-qPCRs using 10-fold serial dilutions. To get the maximum representation of values in the curve, we used for the 10-fold serial dilutions a reference pool made from randomized ten total RNA extracted from NPSs with a Cq value close to 20. The RT-qPCRs were carried out in triplicate according to the specific conditions indicated by the manufacturer and described above. All the RT-qPCRs were performed on the Agilent AriaMx Real-Time PCR System (Agilent Technologies, Reference code. G8830A). We determined the slope by linear regression in GraphPad Prism and defined the required levels for PCR efficiency (E%) > 95% and R-squared (R2) > 0.95. The primer efficiency was calculated according to the formula Efficiency (E%) = (10(-1/Slope))-1) ^∗^ 100 [8]. To determine the detection limit, we select ten samples with Cq values close to 30. Thus, a standard curve determined the minimum detection limit for the TAAG RT-qPCR kit for each probe's amplification. The experimental TAAG RT-qPCR kit LoD was established based on the last dilution of all the triplicates amplified. We also considered the R2 (intended as a goodness-of-fit measure for linear regression) and the probe efficiency (closer to 100%, intended 100% as the optimum probe efficiency value). The experimental LoD was compared with the maximum Cq value suggested by the manufacturer and detailed in the kit brochure.

### 2.6. SARS-CoV-2 Variants Detection

The detection of different punctual mutations (HV69/70del; N501Y; P681H; E484 K; K417 N/T) was made by the AccuPower® SARS-CoV-2 Variants ID Real-Time RT-PCR kit (Bioneer Reference code: SMVR-2112) according to manufacturer's recommendations with some modifications. In brief, 5 *μ*l of RNA extracted from the NPS samples were used for each reaction. The Bioneer Exicycler™ 96 Realtime Quantitative Thermal Block (Bioneer Co., Reference code: A-2060-1) was used for this assay. Data were extracted using the Bioneer Exicycler™ 96 software.

### 2.7. Ethics Statement

All the experimental procedures included in this study were authorized by the Ethical Committee of the University of Santiago of Chile (No. 226/2021) and the Scientific Ethical Committee of the Central Metropolitan Health Service, Ministry of Health, Government of Chile (No. 370/2021), and following the Chilean law in force.

### 2.8. Data Representation and Statistical Analysis

Paired two-sided student's *t*-test was used for the Cq and RFU analysis of the reference gene. A one-way paired ANOVA test was used for the viral gene to analyze the differences between RFU and Cq values. Statistical software GraphPad Prism 8 was used to analyze and graph the data. A *p* < 0.05 was considered statistically significant.

## 3. Results

**Figure 1:**
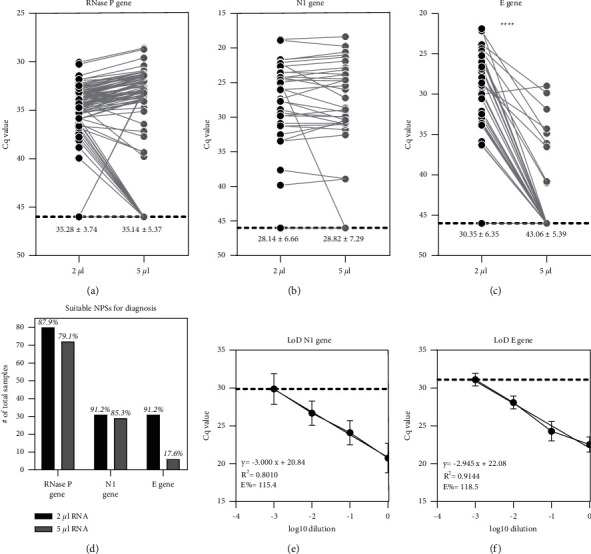
Determination of total RNA extracted from nasopharyngeal swab samples (NPSs) loading volume and the limit of detection (LoD) of SARS-CoV-2 probes using TAAG RT-qPCR kit. The upper section of the figure represents the Cq value paired comparison for total RNA extracted from NPSs loading volume determination. The analysis was made from the same NPSs using the manufacturer's recommended volume of 5 *μ*l and 2 *μ*l of total RNA extracted. In the graphs, the comparison was made for (a) RNase P internal reference, (b) N1, and (c) E viral gene probes. Each spot is a different analyzed sample for each volume condition (2 *μ*l; 5 *μ*l). On (a-c), the mean ± standard deviation (mean ± SD) for all the samples evaluated is indicated for each total RNA volume condition (2 *μ*l; 5 *μ*l). The line linking the spots indicate the paired result obtained for the same sample assessed using the two different volume conditions. Samples with Cq = 46 denote no amplification (indicated in the graph by a black broken line). For statistical analysis, paired two-sided student' *t*-test was applied (*n* = 80 random NPSs, *n* = 31 N1 gene, and *n* = 31 E gene random positive SARS-CoV-2 NPSs, ^*∗∗∗∗*^*p* < 0.05). The lower section of the figure represents (d) the number of amplified samples using TAAG probes loading 2 and 5 *μ*l of total RNA extracted. Limit of detection (LoD) for (e) N1 gene probe and (f) E gene probe for SARS-CoV-2 detection. The analysis included 10-fold serial dilutions from a reference pool made from randomized ten total RNA NPS-extracted samples with a Cq value close to 20 (previously obtained using the Thermo Fisher kit). For the LoD determination, a linear regression was performed.

We evaluated the amplification profile using 2 and 5 *μ*l (the last volume recommended by the manufacturer) of total RNA extracted from 91 random NPSs. Thirty-four samples were previously diagnosed as SARS-CoV-2 positive by the Thermo Fisher kit. Importantly, for the RNase P internal reference gene probe (Figure 1(a)), no significant differences were observed in the mean Cq value of the samples when 2 *μ*l (35.28 ± 3.74) and 5 *μ*l (35.14 ± 5.37) of total RNA were loaded. This implies that the 87.9% (80/91) and 79.1% (72/91) samples, respectively, showed amplification with a Cq value ≤ 38 (considered as a suitable sample for analysis, according to manufacturer's instructions) (Figure 1(d)). For the N1 viral gene probe (Figure 1(b)), no significant differences were observed between their mean Cq values when 2 *μ*l (28.14 ± 6.66) and 5 *μ*l (28.82 ± 7.29) of total RNA were loaded. However, 91.2% (31/34) and 85.3% (29/34) of samples evaluated showed amplification with a Cq value ≤ 37 respectively, according to the manufacturer's recommendation for a positive SARS-CoV-2 sample. For the E viral gene probe (Figure 1(c)) significant differences were observed between their mean Cq values when 2 *μ*l (30.35 ± 6.35) and 5 *μ*l (43.06 ± 5.39) of total RNA were loaded. This implied that 91.2% (31/34) and 17.6% (6/34) of the samples registered amplification with a Cq value ≤ 37 (set following the manufacturer's recommendation for a positive SARS-CoV-2 sample) (Figure 1(d)). Based on these results and previous antecedents for other RT-qPCR kits evaluated (Santibáñez et al., 2021), we defined 2 *μ*l as the chosen loading volume for the TAAG RT-qPCR kit.

The limit of detection (LoD) and the efficiency percentage (E%) of the probes for the TAAG RT-qPCR kit were determined, including the N1 (LoD: Cq value = 29.87, equivalent to 1152.2 copies/*μ*l; E% = 115.4%) (Figure 1(e)) and *E* viral gene probes (LoD: Cq value = 31.09, equivalent to 496.26 copies/*μ*l; E% = 118.5%) (Figure 1(f)). The LoD for the RNase P internal reference gene probe of the TAAG kit was not determined due to the lack of amplification when the samples were diluted (data not shown). In this way, the manufacturer's recommended criteria for considering a sample as a suitable sample for analysis: the Cq value is ≤ 38 for the RNase P reference gene probe. The manufacturer recommends a Cq value ≤ 37 both for N1 and *E* viral genes to consider the sample with SARS-CoV-2 positive diagnostic.

The same analysis was performed for the Thermo Fisher RT-qPCR kit. Thus, the LoD for the ORF1ab gene was 5.97 copies/*μ*l (Cq = 37.15), 6.34 copies/*μ*l for the N gene (Cq = 36.63); 10.28 copies/*μ*l for the S gene (Cq = 35.72), and 3.51 copies/*μ*l for the RNase P reference gene probe (Cq = 37.09) (Supplementary Figures 1 and 2).  Table 1 summarizes the LoDs for each RT-qPCR kit evaluated.

### 3.1. Amplification of Internal Reference and Viral Gene Probes of TAAG RT-qPCR Kit

**Figure 2:**
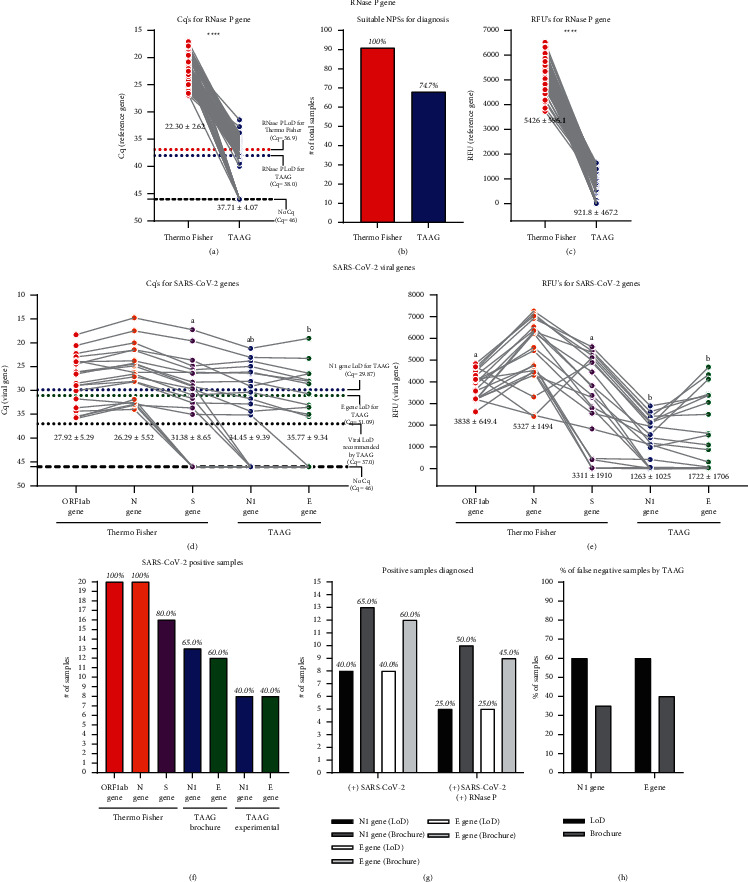
Comparative analysis for the detection of SARS-CoV-2 from random-chosen nasopharyngeal swab samples (NPSs) using Thermo Fisher and TAAG RT-qPCR kits. The comparison was made from the same NPSs using the previously optimized volume of 2 *μ*l of total RNA extracted. The upper section of the figure represents the analysis for the amplification of RNase P internal reference gene probes from 91 random NPSs. (a) Cq value for internal reference RNase P gene probe amplification using Thermo Fisher and TAAG RT-qPCR kits (*n* = 91 NPSs). In graph, the horizontal red-dotted line represents the LoD for RNase P reference gene probe (Cq = 36.9) by Thermo Fisher kit, and the horizontal blue-dotted line represents the maximum recommended Cq value (Cq = 38) by the manufacturer of suitable sample for diagnosis using TAAG RT-qPCR kit RNase P reference gene probe. Samples with Cq = 46 denote no amplification (indicated in the graph by a black broken line). (b) Summary of the percentage of suitable NPSs for diagnosis using the internal reference RNase P gene probe (detailed on (a)) by both RT-qPCR kits. (c) RFU value from the same samples analyzed on (a). Samples with a Cq value > 38 were considered as not suitable according to manufacturer's recommendations. For statistical analysis, a paired two-sided student's *t*-test was applied (*n* = 91 random NPSs, ^*∗∗∗∗*^*p* < 0.05). The lower section of the figure represents the analysis for the amplification of SARS-CoV-2 viral gene probes from 91 random NPSs. Paired comparison for (d) Cq and (e) RFU values from viral gene probe amplification using Thermo Fisher (ORF1ab, N, and S gene probes) and TAAG (N1 and E gene probes) kits (*n* = 20). In graph (d), the horizontal colored-dotted lines blue and green, respectively, represent the experimental LoD for N1 gene (Cq = 29.87) and E gene (Cq = 31.09) probes of TAAG RT-qPCR kit. The horizontal black dotted line represents the maximum Cq value (Cq = 37) recommended by the manufacturer for positive sample diagnosis using TAAG RT-qPCR viral probes. Samples with Cq = 46 denote no amplification (indicated in the graph by a black broken line). For statistical analysis, a paired one-way ANOVA-test with multiple comparison test analysis applied. Lowercase letters above each probe's spot columns indicate which probes do not show significant differences between them. (*n* = 20 NPSs, ^∗∗∗∗^p < 0.05). In graphs (a), (c), (d), and (e), the mean ± standard deviation (mean ± SD) is indicated for the viral gene probe amplification obtained for all the samples evaluated. The line linking the spots indicated the paired result obtained for the same sample assessed by both RT-qPCR kits. (f) Summary of the number of positive NPSs detected using Thermo Fisher (ORF1ab, N, and S probes) and TAAG RT-qPCR kit (N1 and E viral gene probes applying brochure or experimental LoD criteria ((n) = 20). (g) Summary of the number of positive NPSs diagnosed using TAAG RT-qPCR kit considering the RNase P internal control amplification. In the graph, “(+) SARS-CoV-2” represents a positive virus diagnosis, and “(+) RNase P” represents the amplification of the internal reference gene. (h) Summary of the percentage of false negative NPSs diagnosed by TAAG RT-qPCR kits applying brochure or experimental LoD criteria.

Once the LoD for the TAAG kit was determined, 91 NPS random samples were analyzed and compared their amplification parameters (Cq and RFU values) between the Thermo Fisher and TAAG RT-qPCR kits. The paired comparison of the Cq values of the RNase P internal reference gene probe (Figure 2(a)) showed a significant difference between both RT-qPCR kits. The samples analyzed by the Thermo Fisher kit showed a mean Cq value of 22.30 ± 2.62, while the TAAG kit showed 37.71 ± 4.07. Importantly, all these samples showed amplification for the RNase P internal reference gene probe, making them suitable for diagnostic when analyzed by the Thermo Fisher kit (Figure 2(b)). However, when TAAG analyzed the same samples, only 74.7% (68/91) of them could be considered suitable samples for diagnosis because their Cq values were ≤38 ( the maximum recommended Cq value for sample diagnosis). The paired comparison of RFU values obtained for both kits also showed significant differences (Figure 2(c)), with mean values of 5426 ± 596.1 and 921.8 ± 467.2 for the Thermo Fisher and the TAAG RT-qPCR kits, respectively.

Also, the comparison of the viral genes of both kits was assessed. The paired comparison of the mean Cq values of viral gene probes of both RT-qPCR kits (Figure 2(d)) showed a significant difference, except between the S gene probe (31.38 ± 8.65) with the N1 gene probe (34.45 ± 9.39) and the *E* gene probe (35.77 ± 9.34) from the TAAG kit. The paired comparison of the RFU values showed significant differences between any of the viral gene probes between both RT-qPCR kits (Figure 2(e)). No differences in the mean RFU value were registered between ORF1ab (3838 ± 649.4) and S viral gene probes (3311 ± 1910) for the Thermo Fisher kit. The same case was also reported between N1 (1263 ± 1025) and *E* viral gene probes (1722 ± 1706) for the TAAG kit (Figure 2(e)).

When comparing the number of samples with a positive diagnosis for SARS-CoV-2 based on the viral gene amplification, it was observed that the number of positive samples detected by each kit varies worryingly. The Thermo Fisher kit showed amplification for 100% (20/20) of positive samples for viral ORF1ab and N gene probes (Figure 2(f)), while for the viral S gene probe, it only detected 80% (16/20) of those positive samples. In the case of the TAAG kit, the viral gene probes N1 and *E* were noticed in only 65% (13/20) and 60% (12/20) of those positive samples, respectively, when recommended manufacturer criteria were applied (Figure 2(f), TAAG brochure). However, when the experimentally determined LoD was used (Figure 2(f), TAAG experimental), both viral gene probes detected only 40% (8/20) of those positive samples.

When the criteria of Cq values recommended by the manufacturer for viral genes (Cq ≤ 37) and the internal reference gene (Cq ≤ 38) are applied to ensure the diagnostic of samples, the situation becomes more complex for the TAAG kit. For N1 and *E* probes, only 50% (10/20) and 45% (9/20), respectively, of positive SARS-CoV-2 samples were reported when the RNase P internal reference gene showed amplification (Figure 2(g)). However, when the experimentally determined LoD was applied, only 25% (5/20) of the samples were reported as positive for SARS-CoV-2 for TAAG viral gene probes (Figure 2(g)). As a consequence, the analysis with the N1 viral gene probe (Figure 2(h)) showed a 35% (7/20) and 60% (12/20) false-negative diagnosis when the manufacturer's recommendation (Cq ≤ 37) and experimental LoD (Cq ≤ 29.87) were applied, respectively. Similar results were obtained for the *E* viral gene probe (Figure 2(h)), 40% (8/20) and 60% (12/20) of false-negative diagnosed samples applying the manufacturer's recommendation (Cq ≤ 37) and experimental LoD (Cq ≤ 31.09), respectively.

**Figure 3:**
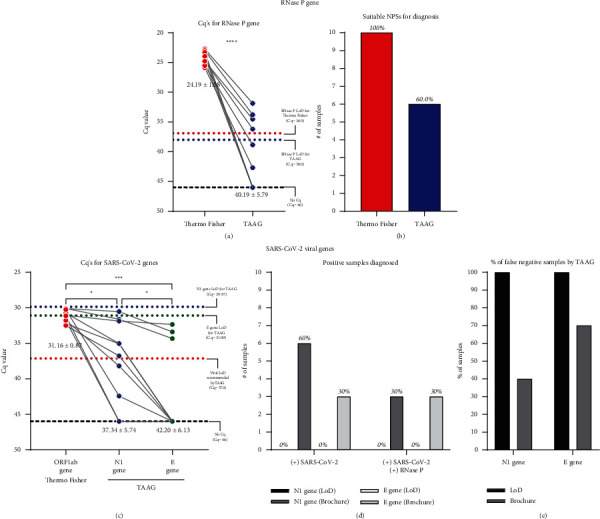
Comparative analysis between Thermo Fisher and TAAG RT-qPCR kits with a known Cq value close to 30 (for ORF1ab gene probe, Thermo Fisher). The comparison was made for the same NPSs using the previously optimized volume of 2 *μ*l of total RNA extracted. The upper section of the figure represents the analysis for the RNase P internal reference gene probe amplification from 10 NPSs previously diagnosed as SARS-CoV-2 positive by the Thermo Fisher RT-qPCR kit with a Cq value close to 30. (a) Paired comparison of Cq value of RNase P internal reference gene probes from Thermo Fisher and TAAG kits. In graph, the horizontal red-dotted line represents the LoD for RNase P reference gene probe (Cq = 36.9) by Thermo Fisher kit, and the horizontal blue-dotted line represents the maximum recommended Cq value (Cq = 38) by the manufacturer for suitable sample diagnosis using TAAG RT-qPCR kit RNase P reference gene probe. Samples with Cq = 46 denote no amplification (indicated in the graph by a black broken line). For statistical analysis, a paired two-sided student's t-test was applied (*n* = 10 NPSs, ^*∗∗∗∗*^*p* < 0.05). (b) Number of suitable samples diagnosed with a Cq value within the detection range of the internal reference gene probes. The lower section of the figure represents the analysis for the viral gene probe amplification from 10 NPSs previously diagnosed as SARS-CoV-2 positive by the Thermo Fisher RT-qPCR kit with a Cq value close to 30. (c) Paired comparison of Cq value of viral gene probes from Thermo Fisher (ORF1ab viral gene probe) and TAAG (N1 and E viral gene probes) kits. In graph, the horizontal colored-dotted lines blue, green, and red, respectively, represent the experimental LoD for N1 gene (Cq = 29.87), E gene (Cq = 31.09), and ORF1ab gene (Cq = 37.14) viral gene probes. Samples with Cq = 46 denote no amplification (indicated in the graph by a black broken line). For statistical analysis, paired one-way ANOVA-test with multiple comparison test analysis was applied. (n = 10 NPSs, ^*∗∗∗∗*^*p* < 0.05). In graphs (a) and (c), the mean ± standard deviation (mean ± SD) is indicated for the viral gene probe amplification obtained for all the samples evaluated. The line linking the spots indicate the paired result obtained for the same sample assessed by both RT-qPCR kits. (d) Summary of the number of positive NPSs diagnosed using TAAG RT-qPCR kit considering the RNase P internal control amplification. In the graph, “(+) SARS-CoV-2” represents a positive virus diagnosis, and “(+) RNase P” represents the amplification of the internal reference gene. (e) Summary of the percentage of false negative NPSs diagnosed by TAAG RT-qPCR kits applying brochure or experimental LoD criteria.

Because of the worrying percentage of false negatives observed for the TAAG kit (Figure 2(h)), we evaluated the performance of the TAAG kit with ten previously diagnosed SARS-CoV-2 positive samples with Cq values close to 30 using the Thermo Fisher kit (Figure 3). The amplification of the RNase P internal reference gene probe (Figure 3(a)) showed significant differences between the mean Cq value of both RT-qPCR kits (Cq_Thermo Fisher_ = 24.19 ± 1.08; Cq_TAAG_ = 40.19 ± 5.79). The RNase P internal reference gene probe amplification showed all the samples suitable for diagnosis by the Thermo Fisher kit (Figure 3(b)). However, when TAAG analyzed the same samples, only 60% (6/10) of these could be considered suitable samples for diagnosis because their Cq value was ≤ 38, the maximum Cq value recommended for diagnosis. When the amplification of the viral genes was analyzed, we registered differences between the mean Cq value of ORF1ab (31.16 ± 0.87), N1 (37.34 ± 5.74), and *E* (42.20 ± 6.13) viral gene probes (Figure 3(c)). The percentage of these SARS-CoV-2 positive samples diagnosed by the viral gene probes of the TAAG kit (Figure 3(d)), following the manufacturer's recommendations, was 60% (6/10) and 30% (3/10) for N1 and *E* viral gene probes, respectively. When the criteria of internal reference gene amplification (Cq ≤ 38) ((+) RNase P) were applied, both viral probes showed 30% (3/10) of samples as positive for SARS-CoV-2 (Figure 3(d)). This implies for the TAAG kit a 40% (4/10) and 70% (7/10) false-negative samples were diagnosed for N1 and *E* gene probes, respectively (Figure 3(e)). However, no one positive sample was detected when the experimentally determined LoD was applied to TAAG viral gene probes (Figure 3(d)). Thus, 100% (10/10) of false-negative diagnoses were recorded using the TAAG RT-qPCR kit on samples previously detected with a Cq value close to 30 by the Thermo Fisher kit (Figure 3(e)).

### 3.2. Impact of the TAAG Diagnostic Kit on the Detection of SARS-CoV-2 Variants

**Figure 4:**
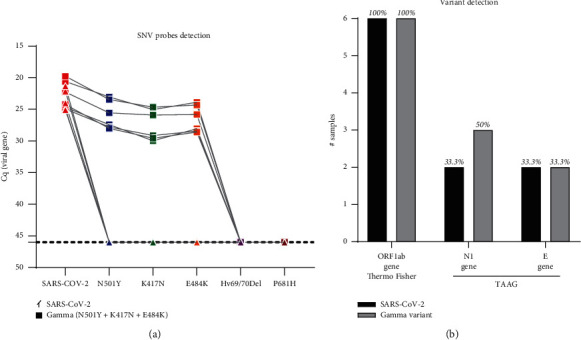
Impact of the TAAG diagnostic kit on the detection of SARS-CoV-2 variants. Twelve previously diagnosed SARS-CoV-2 positive samples by Thermo Fisher kit were randomly chosen and analyzed for the presence of SARS-CoV-2 variants by RT-qPCR. (a) Cq values for detected SNV probes associated with SARS-CoV-2 variants (N501Y, K417 N/T, E484 K, Hv 69/70 del, and/or P681H). When only the original SARS-CoV-2 variant was detected, it was denoted with triangles (▲). When the Gamma variant sample (N501Y, K417 N/T and E484 K probes positive amplification) was detected, it was denoted with squares (■). The other six samples showed no amplification for any of the SNV probes tested. (b) Summary of the diagnostic impact of the TAAG (N1 and E genes) and Thermo Fisher (ORF1ab gene) on the detection of SARS-CoV-2 variants.

Twelve previously diagnosed SARS-CoV-2 positive samples by Thermo Fisher kit were randomly chosen and analyzed for the presence of SARS-CoV-2 variants by RT-qPCR. The detection of single nucleotide variations (SNV) was focused on the S gene. Six of these samples showed amplification only for the SARS-CoV-2 probe but not for any SNV probes (SARS-CoV-2(+) SNV(-); denoted with triangles (▲)) (Figure 4(a)). The TAAG N1 and *E* gene probes identified only the 33.3% (2/6) of samples from these six samples (Figure 4(b)). The other six samples showed amplification for SARS-CoV-2 and for the N501Y, K417 N/T, and E484 K SNV probes (SARS-CoV-2(+) SNV(+); denoted with squares (■)) (Figure 4(a)) suggesting that these samples correspond to the SARS-CoV-2 variant of concern (VOC) Gamma. From these six samples, the TAAG N1 gene probe identified only 50% (3/6), whereas the *E* gene probe identified only 33.3% (2/6) (Figure 4(b)).

## 4. Discussion

Currently, multiple RT-qPCR kits are commercially available for SARS-CoV-2 detection. However, they lack appropriate clinical analysis and validation for use in the COVID-19 pandemic. Various studies of comparative analysis and proofs of RT-qPCR kits have previously been reported. For example, comparisons of kits used for COVID-19 diagnosis in Korea showed differences of up to 25% in the sensitivity of detection of SARS-CoV-2 [9]. In China, some kits used have 83% and 94% sensitivity, according to Sansure Biotech Inc. and BioGerm Medical, respectively, both with 100% specificity without cross-reaction in the detection of other viruses [10]. On the other hand, Eberle et al. 2021, analyzed nine commercial RT-qPCR kits used for the diagnosis of COVID-19 in Bavaria, Germany, with differences of up to 50% in detection sensitivity [6]. Also, a study using RNA from cell cultures identified differences in the number of viral copies detected in eleven commercial RT-qPCR kits, which varied between 3.3 and 330 RNA copies, where one also jointly noticed another human coronavirus (MERS), revealing nonspecificity in the diagnosis for SARS-CoV-2 [11]. All these studies strongly support the importance of an in-depth analysis of the performance of the RT-qPCR kits used to control the current pandemic, which translates into better or worse control and traceability of infected patients. However, to date, no study has reported an analysis of the RT-qPCR kits widely used to diagnose SARS-CoV-2 in the Chilean population. Furthermore, none of the previous studies has considered a deep analysis of the RT-qPCR parameters of the cellular internal reference gene (only viral target genes are mainly considered). The amplification of the internal reference gene is essential but underestimated data in an RT-qPCR analysis to ensure the accuracy of negative and positive results in clinical diagnosis [12].

We found that the TAAG kit showed serious difficulties in amplifying the internal and viral reference genes, along with both high Cq and low RFU values. This was reflected in a difference of up to 40% in SARS-CoV-2 detection where the Thermo kit showed a detection efficiency of 100% for N and ORF1ab genes. In addition, the samples analyzed with the Thermo Fisher kit showed RFU values up to 6 times higher than those observed with the TAAG RT-qPCR kit, indicating less inhibition in the RT-qPCR reaction. Based on these data, it is not surprising that the diagnosis of NPSs includes a high number of false-negative diagnoses by the TAAG kit. This is because the TAAG kit has an LoD of over 1000 viral copies/*μ*l for N1 and about 500 viral copies/*μ*l for the *E* gene, which is equivalent to a sensitivity of up to 100 times less compared to the Thermo kit. Fisher. Consequently, the detection of SARS-CoV-2 by the TAAG kit was seriously compromised.

Our report was closely related to that previously described [13] for the Thermo Fisher RT-qPCR kit, which has shown a sensitivity of >96% in detecting SARS-CoV-2. This evidence was also according to other reports showing the optimal performance of the Thermo Fisher kit for the detection of low viral loads (Cq > 30) [13], and in the detection of SARS-CoV-2 in samples without a previous total RNA extraction process (which decreases the amount of free RNA in the sample to be analyzed) [14]. This background confirms the high sensitivity of this kit for the diagnosis of COVID-19. On the other hand, most of the comparative investigations of different RT-qPCR kits for the diagnosis of COVID-19 focus only on the amplification of the SARS-CoV-2 gene, omitting the information of the reference gene, which can invalidate a result if it presents faulty parameters [15–17]. For example other kits use, for example, exogenous internal controls (e.g., EAV; equine arteritis virus, etc.). However, this is only an intrinsic control and is not related to quality and a competent sample [18]. In relative expression analyses, the reference gene is essential to normalize the expression levels of target genes [12]. In diagnosis, it is a solid basis to validate the proper process of total RNA extraction and subsequent amplification of the SARS-CoV-2 genes of interest used (ORF1ab, N, S, *E*, and RdRp). Due to the nonamplification of an internal gene, the results may be invalidated [19].

The comparative analysis between the Thermo Fisher and TAAG RT-qPCR kits revealed differences in the RFU values obtained for the same set of samples, indicating differences in kit performance and specifically in the degree of inhibition for each reaction [20]. A low RFU value could even be mistaken for a background signal [21]. In this way, the incorporation of RFU values analysis supports our results. For this reason, we suggest that it is also a parameter that should be carefully considered to decide if a sample is suitable for issuing a diagnostic result. Therefore, these findings indicate limitations on the use of the TAAG kit for the diagnosis of COVID-19. As a consequence, the misidentification of positive cases also compromises the identification of circulating SARS-CoV-2 variants in the population (like the gamma variant). RT-qPCR kits were developed to detect characteristic SNV associated with SARS-CoV-2 variants. However, samples must be diagnosed as positive using an RT-qPCR kit before this analysis. Therefore, it is essential to avoid using low-sensitivity RT-qPCR kits since this could include false-negative diagnoses and ambiguities in the clinical diagnosis. This is a matter of critical concern, especially at this epidemiological stage of the pandemic with the presence of more contagious variants such as omicron [22].

Our results do not consider the specificity of the analyzed kits. In this regard, some reports indicate cross-reaction with other types of viruses [11], which is an aspect to be considered by the manufacturers of commercial RT-qPCR kits. Thus, we suggest an exhaustive analysis of all commercial kits before they are used for the best control of this and other infectious diseases

## 5. Conclusions

Our results indicate that (1) the TAAG kit shows low Cq values for internal and viral genes of SARS-CoV-2 due to low detection of viral copies/*μ*l . (2) The TAAG RT-qPCR kit negatively impacts the detection for the gamma variant of SARS-CoV-2 compared to the Thermo Fisher kit. (3) The massive use of kits with a high risk of diagnosis of false negatives could jeopardize the application of public policy measures to control the pandemic.

## Figures and Tables

**Table 1:** Summary of limit of detection (Lod, viral copies/µl) for each probe of the analyzed RT-qPCR kits

TAAG RT-qPCR Kit LoD (copies/*μ*l)			Thermo Fisher RT-qPCR Kit LoD (copies/*μ*l)			
RNase P	N1	E	RNase P	ORF1ab	N	S
n/d	1152.2	496.26	3.51	5.97	6.34	10.28

## Data Availability

The data used to support the findings of this study may be released upon direct reasonable request to the corresponding author, who can be contacted at e-mail felipe.reyes.l@usach.cl and felipe.reyes@uab.cat.

## Supplementary Materials

Supplementary Materials Supplementary Figure 1: probe efficiency and limit of detection (LoD) of Thermo Fisher RT-qPCR. The efficiency and LoD of Thermo Fisher probes were determined using 10-fold serial dilutions from ten positive samples. The LoD for the (*A*) ORF1ab gene probe was 5.97 copies/*μ*l (Cq = 37.15); (B) 6.34 copies/*μ*l for the N gene probe (Cq = 36.63); (C) 10.28 copies/*μ*l for the S gene probe (Cq = 35.72); and (D) 3.51 copies/*μ*l for the RNase P reference gene probe (Cq = 37.09). Supplementary Figure 2: standard curve for Thermo Fisher RT-qPCR probes. Standard curves for (A) ORF1ab gene, (B) N gene, (C) S gene, and (D) RNase P gene probes, developed from 10-fold serial dilutions of the synthetic positive control of the Thermo Fisher RT-qPCR kit. In the graph, the dotted black line denotes the LoD for each probe.
