# Expression of Concern on “The Comparative Analysis of Two RT-qPCR Kits for Detecting SARS-CoV-2 Reveals a Higher Risk of False-Negative Diagnosis in Samples with High Quantification Cycles for Viral and Internal Genes”

**DOI:** 10.1155/2023/9798013

**Published:** 2023-01-19

**Authors:** Canadian Journal of Infectious Diseases and Medical Microbiology

*Canadian Journal of Infectious Diseases and Medical Microbiology* would like to express concern with the article titled “The Comparative Analysis of Two RT-qPCR Kits for Detecting SARS-CoV-2 Reveals a Higher Risk of False-Negative Diagnosis in Samples with High Quantification Cycles for Viral and Internal Genes” [1]. A concern has been raised regarding a potential conflict of interest in this article and possible errors in the methodology used. In light of this concern, the publisher is investigating further and will revisit this article with any necessary post-publication amendments.

## References

[1] Luraschi R., Barrera-Avalos C., Vallejos-Vidal E. (2022). The Comparative Analysis of Two RT-qPCR Kits for Detecting SARS-CoV-2 Reveals a Higher Risk of False-Negative Diagnosis in Samples with High Quantification Cycles for Viral and Internal Genes. *The Canadian Journal of Infectious Diseases & Medical Microbiology*.

