# Supplementary material for: The Comparative Analysis of Two RT-qPCR Kits for Detecting SARS-CoV-2 Reveals a Higher Risk of False-Negative Diagnosis in Samples with High Quantification Cycles for Viral and Internal Genes
Source: Can J Infect Dis Med Microbiol. 2022 Jul 5;2022:2594564. doi: 10.1155/2022/2594564 (PMC9259548; doi:10.1155/2022/2594564)
Supplement: Supplementary Materials — Supplementary Figure 1: probe efficiency and limit of detection (LoD) of Thermo Fisher RT-qPCR. The efficiency and LoD of Thermo Fisher probes were determined using 10-fold serial dilutions from ten positive samples. The LoD for the (A) ORF1ab gene probe was 5.97 copies/μl (Cq = 37.15); (B) 6.34 copies/μl for the N gene probe (Cq = 36.63); (C) 10.28 copies/μl for the S gene probe (Cq = 35.72); and (D) 3.51 copies/μl for the RNase P reference gene probe (Cq = 37.09). Supplementary Figure 2: standard curve for Thermo Fisher RT-qPCR probes. Standard curves for (A) ORF1ab gene, (B) N gene, (C) S gene, and (D) RNase P gene probes, developed from 10-fold serial dilutions of the synthetic positive control of the Thermo Fisher RT-qPCR kit. In the graph, the dotted black line denotes the LoD for each probe. [file 2594564.f1.zip › 2594564.f1/Supplementary Figure Captions.docx]

**SUPPLEMENTARY FIGURE CAPTIONS**

**Supplementary Figure 1. Probe efficiency and limit of detection (LoD) of Thermo Fisher RT-qPCR.** The efficiency and LoD of Thermo Fisher probes were determined using 10-fold serial dilutions from ten positive samples. The LoD for (A) ORF1ab gene probe was 5.97 copies/µl (Cq= 37.15); (B) 6.34 copies/µl for N gene probe (Cq= 36.63); (C) 10.28 copies/µl for S gene probe (Cq= 35.72); and (D) 3.51 copies/µl for RNase P reference gene probe (Cq= 37.09).

**Supplementary Figure 2. Standard curve for Thermo Fisher RT-qPCR probes.** Standard curves for (A) ORF1ab gene, (B) N gene, (C) S gene, and (D) RNase P gene probes, developed from 10-fold serial dilutions of the synthetic positive control of the Thermo Fisher RT-qPCR kit. In the graph, the dotted black line denotes the LoD for each probe
